# Anemia

**Published:** 1844-09

**Authors:** John McLean

**Affiliations:** Professor of Materia Medica and Therapeutics in the Rush Medical College; Jackson, Mich.


### ILLINOIS

### MEDICAL & SURGICAL JOURNAL.

VOL. I. SEPTEMBER, 1844. NO. 6

### *Anemia.* By John McLean, Professor of Materia Medica and Therapeutics in the Rush Medical College.

(*Continued from page* 79.)

*Causes continued.—Why the periods of Gestation and Lactation are more particularly subject to Anemia.—Case of Mrs. S., and Post Mortem Examination.—Treatment.*

When subjected to the malarious influence, if a periodical disease is not clearly developed, there may be produced such symptoms as anorexia, indigestion, foul tongue, increased frequency of the pulse, with a sense of languor, and general debility; erratic pains in different parts of the body, achings about the joints, and a variable sensation of temperature. And if the true cause of the difficulty is overlooked, it may exist, operate upon and undermine for a long time the powers of the constitution.

The greater frequency of *anemia* in western localities, than in the older settled portions of our country, cannot be owing to innutritious or scanty food, open or badly constructed dwellings, or western hardships; for it is also more frequent among that class who are not subjected to any of these causes. Therefore, I think, the most probable cause that we can assign for its greater prevalence in such localities, is the operation of that agent, which is more particularly confined to new countries, viz: *marsh miasmata.*

Why is anemia principally confined to females during the periods of gestation and lactation?

From my own observation, and from information obtained from others who have had extensive experience in malarious districts, I am led to believe, that in the above conditions, the malarious agent is less marked by its peculiarity of producing periodical diseases. And if this be so, may not the true cause of the existing difficulty be oftener overlooked than it otherwise would be, and the system left to suffer under its deleterious influence? I am aware that many differ from the opinion here expressed, and think the periodical character of the diseased action is less evident.

But still further, the large demands made of the system for the growth of the fœtus during gestation; and afterwards, the daily and almost constant waste that is made upon the essential elementary constituents of the blood, by the abstraction of the milk for the nourishment of the infant, are causes well calculated, in the feeble and sickly, to hasten on the state of *anemia.* There is a close resemblance between the composition of milk and blood. Milk, in its composition, is more nearly allied to blood than any of the secreted fluids. The following table from "Carpenter's Human Physiology," will show the parallelism of their several ingredients:

| | *Blood.* | *Milk.* | |
|---|---|---|---|
| Coagulum. | Fibrine, | Caseine, | Cream. |
| | Red particles. | Butyraceous matter, | |
| Serum. | Albumen, | Caseine, | Skim milk. |
| | Alcoholic Extractive, | Alcoholic Extractive, | |
| | viz: Lactates, | viz: Lactates and Lactic Acid, | |
| | Aqueous Extractive, | Aqueous Extractive with | |
| | Albuminate of Soda, | Sugar of milk, | |
| | Alkaline Salts, | Alkaline Salts, | |
| | Fatty matter, | Fatty matter, | |

The chief proximate animal matters have nearly the same composition, and may be regarded as definite compounds with proteine. Phosphorous and sulphur in proportions slightly varying, combined with proteine form fibrine, albumen and caseine.

The following ultimate analysis was lately executed in the Laboratory of Liebig, and exhibits the elements of fibrine and albumen, proximate principles of blood; and of caseine a proximate principle of milk.

| | BLOOD. | | MILK. |
|---|---|---|---|
| | *Fibrine.* | *Albumen.* | *Caseine.* |
| Carbon, | 54.56 | 54.84 | 54.96 |
| Nitrogen, | 15.72 | 15.83 | 15.80 |
| Hydrogen, | 6.90 | 7.09 | 7.15 |
| Oxygen, Phosphorous, Sulphur, | 22.82 | 22.24 | 22.09 |
| | 100.00 | 100.00 | 100.00 |

The correspondence between the red particles and the butyraceous matter is less evident; but there are some points of resemblance between them. The saline matters contained in milk and blood are nearly identical. There is perhaps a greater proportion of the phosphates of lime and magnesia in the former than in the latter. The correspondence between the rest of the table is perfectly evident, and needs no comments. Now it is easy to conceive why such females should become anemic, as have for a long time been subject to the slow poison of marsh miasmata, and feeble health; and in addition to this, whose sanguine humor is drawn largely upon for the supply of the fœtus, and afterwards, during the period of lactation, by that fluid, which so much resembles the blood in its composition.

*Case of Mrs. S.*—I do not give this because it had any striking peculiarities, but because it was a well marked case of the kind, and one in which I was privileged with a *post mortem* examination. Mrs. S., sometime in the spring or early part of the summer of 1843, removed from the State of Vermont to the Territory of Wisconsin, where she resided about four months. Thence she removed to this place, (Jackson, Mich.) About the middle of January, 1844, she was delivered of her second, a healthy child. She was naturally of a rather delicate constitution; and at this time was pale and feeble; and for some weeks after her confinement was under medical treatment. Her child, she continued to nurse until about the 1st of April. About the middle of April, I was called to see her and found her in the following condition: the prolabia, instead of a florid hue, were pale and presented almost a bloodless appearance. The tongue and inside of the mouth (except in the vicinity of ulcers) wore nearly the same aspect. The face was extremely white, and seemed as if entirely destitute of red blood. On the tongue and inside of the cheeks were small and irritable ulcers. These first made their appearance about one week after the birth of her child. She had a diarraœa which would leave her, and return at irregular intervals. Indigestion was quite a troublesome symptom, and at times the irritability of the stomach was quite great. For the most part there was anorexia; but at times there was considerable appetite; but food of almost any kind was apt to create unpleasant symptoms. For a time she had an irritative cough, which at nights was peculiarly distressing. She was extremely weak and irritable, and was much of the time confined to the bed. Pulse 130 a minute—small and feeble. After visiting her some four or five times, I discovered that she was better and worse on alternate days. These variations were very slight, and she never had any greater symptoms of intermittent than this. About 6 grs. of quinia, in divided doses, were now administered daily. Under the use of this, the periodical symptom soon gave way and she appeared much better. After a few days the *quinia* created so much irritation of the stomach that it could not be taken. It was now administered in smaller doses, and with sulphate of morphia, to allay the irritability; but unless greatly under the influence of morphia, the irritation it produced was so great, that its use could not be continued. The quinia was now withheld and other tonics resorted to; among which were some of the preparations of *iron;* but not being attended with any benefit, they were likewise withdrawn. Stimulants were also tried but the effect was unfavorable. Notwithstanding the free use of morphia, she had frequent spells of vomiting which greatly reduced her strength, and at times were very distressing. Both in diet and medicine, the greatest care was necessary not to allow anything that would hasten on, or aggravate this distressing symptom. The local applications to the ulcers in the mouth were such as are usual in such cases. The *commercial ferrocyanate of iron* was now administered in doses of 4 grs. each,—three times daily. This, instead of creating irritation, greatly allayed it—so much so, that the morphia which was taken to allay the irritability, was now almost entirely left off. Under the use of this, a highly nitrogenized diet and occasional slight laxatives, she improved beyond my anticipations. After being under this course two weeks, the pulse became reduced to 110, the appetite and strength were improved, the diarrhœa lessened, and the cough much better. On the 14th of May, she changed her boarding place, for another, forty or fifty rods distant. The fatigue attending this change operated rather unfavorably; but notwithstanding, she appeared better than previous to the use of the quinia and ferrocyanate of iron. On the 25th day of May, she walked down a pair of stairs, and to a carriage, in front of the house, and rode a distance of one mile and a half. The fatigue produced by this was great. She now continued to fail daily. In three or four days time she was confined to her bed. The feet became œdematous; the eyelids and face swollen, and the ulcers large and fetid. Extreme exhaustion was now present, and she gradually sunk away, and died on the 8th of June.

*Post mortem Appearances.*—The organs within the thorax were first examined, but they presented no abnormal appearances except their almost bloodless condition. On dividing some of the large vessels, the fluid which escaped presented the appearance of but slightly colored serum. On opening the heart, it was found entirely destitute of any coagula. In short, the coloring matter and fibrine of the blood were almost entirely absent. The internal surfaces of the *stomach*, *duodenum*, *jejunum*, *ileum*, *and colon* were next examined, but no organic disease could be found; the only unnatural appearance presenting itself being that of a pale and bloodless condition. The *liver*, *spleen*, and *uterus* were also free from any organic disease; but their anemic appearance corresponded with that of the general system. Both internally and externally, bloodlessness and paleness were most conspicuously marked. The *post mortem* appearances gave us no positive light on the true cause of *anemia;* but it gave this negative evidence, that it was not owing to any perceptible altered organic structure.

*Treatment.*—From what has already been said, the plan of treatment about to be recommended, can be anticipated. The first object should be to remove, as far as possible, all known existing causes. If living in a *malarious* district, the patient should, if practicable, change it for a more salubrious locality. If lactation is kept up, it should immediately be discontinued; for while a large healthy child is daily drawing support from the little remaining stock of blood, the cure is much delayed, if not entirely prevented. If the mode of living is bad and the diet poor, they should be exchanged for better. There is a forming stage when the anemic symptoms are not very distinctly marked. This period should be closely watched; for it is the most favorable time to arrest the progress of the disease. If the attending symptoms of this stage are such as to induce the belief that the patient is laboring under the influence of the malarious poison, the treatment should be accordingly. If there is a feverish state of the system, anorexia, foul tongue, and biliary derangement, some mercurial preparation should be given to correct the secretions. The quantity to be used, and how combined with other articles, and the time of its continuance, should be judged of by the symptoms present and the effects produced. After this, or even in connexion with it, sulphate of quinia should be given, if there are no symptoms present to forbid its use. At first, for a few days, it may be given quite freely; afterwards in smaller and less often repeated doses; say 1 gr. morning, noon, and evening. In some cases I have given the quinia without any preparatory treatment, and with a happy effect. I have lately administered it in a few incipient cases, and the benefit derived was prompt and decisive. In recent conversation with a highly respectable physician, I was informed that he had used this article in cases of this kind with marked benefit; that he had given it merely for its tonic properties, without any regard to its specific operation upon the malarious poison; and that upon reflection, he recollected of having used it with decided advantage, where other tonics were useless or even injurious.

Some of the ferruginous preparations may be given, alternately, with the quinia, and even continued for some time after the latter is withheld. In those cases attended with much irritability, the ferrocyanate of iron will be found to be a valuable preparation. If the commerical *ferrocyanate* is used, it should be administered in doses of from 3 to 6 grs., three times a day. The dose of the pure article should be smaller. I will here make the suggestion, that in the state of great irritability of the digestive organs, already referred to, the combination of small doses of quinia with the ferrocyanate of iron might be used with advantage: and I regret that, after having twice failed in the use of quinia, in the case of Mrs. S., I did not make another attempt with this ferruginous preparation, which, for a time she seemed to have taken with a happy effect.

I have used, in many cases, a pill composed of equal parts of gum aloes, rad. podophyllum peltatum, and rad. sanguinaria canadensis. A sufficient quantity of these, given every evening, to procure moderate evacuation from the bowels, on the following day, I have found to do much towards arresting the diarrhœa, and creating a more healthy action of the digestive organs. If the diarrhœa is excessive, and disposed to continue, it may be checked by act. lead and opium; but until there is a more healthy action of the general system, but especially of the digestive organs, it is likely soon to return. Many local applications have been used for the ulcers in the mouth. When the ulcers are irritable and painful, I have derived much benefit from adding a few drops of the tincture of croasote to a small quantity of water, and directing this to be held a short time in the mouth. The nitrate of silver makes a good wash, and I generally prefer it to the many others which have been used. However, applications of this kind afford but temporary relief, until there is brought about a more healthy action of the general system. The diet should be light and nutritious, and composed principally of such articles as contain a large proportion of *nitrogen.*

This article might be extended to a much greater length; but if what has already been said, will be the means of directing the attention of the profession more to this disease, and of inducing them to give the result of their observation, my object will be fully attained.

Jackson, Mich., August, 1844.

